# Case of Puerperal Peritonitis

**Published:** 1815-06

**Authors:** Thomas Atkinson

**Affiliations:** of Kilham, Yorkshire.


*For the Medical and Physical Journal.*

### *Case of Puerperal Peritonitis;* by Mr. THOMAS ATKINSON, of Kilham, Yorkshire.

A. T. was, on the 13th of March, delivered of her first child, after a short and natural labour, the placenta separating without difficulty. She appeared to recover very well until the morning of the 15th, when she was affected with slight shivering, succeeded by pain in the forehead, over the orbits, and also in the abdomen: this increased rather rapidly, and was so violent, at intervals, over the whole belly, that for several hours her cries were heard round the neighbourhood. I was called to her about four in the afternoon. The abdomen was then considerably swollen and tense. The least pressure could not be borne, especially about the navel. Pulse 126, rather full; tongue white; skin very hot; cheeks occasionally flushed, with distressing pain over the eyes, which sometimes removed to the side of the head. The secretion of milk did not appear impaired, nor the lochia much obstructed, but of very bad smell. She could not turn on her side without screaming, and seemed to half breathe from the pain excited by inspiration; had not been able to evacuate the urine since morning; did not feel much sickness, or any inclination to vomit. About 18 or 20 ounces of blood (very buffy) were drawn from the arm, which gave some relief. The bowels being bound, an oily enema was administered, which brought away a quantity of hardened fæces. Half an ounce of the Ol. Ricini was ordered every two hours till it operated. A

 fomentation fomentation was used to the body. She was easier some hours, but the pain returned during the night most violently, and continued till the morning of the 16th, when I visited her, and took away about 18 ounces more blood, with very great relief to the abdominal pain. The fomentation was also repeated, and a saline mixture ordered. She had had several stools during the night, from the castor oil, which were brown and frothy, having, as the nurse described it, very much the appearance of yeast. I saw her again in the afternoon of the same day, and drew off by the catheter above a quart of urine. The pain in the head and abdomen much easier; was quite sensible; the pulse smaller, about 135; tongue much furred. I now determined (on a recurrence of the symptoms) to try the Sp. of Turpentine, recommended by Dr. Brenan in his late most valuable paper in your excellent Journal. Towards evening her pain returned violently, and for some hours she appeared in a state of stupor and insensibility, occasionally shrieking. About eight o'clock, about two drams of the Sp. Terebinth. were given her in some weak mint water: within twenty minutes she felt very great relief, and called out for a repetition of the dose, which was given her four times, at an interval of four hours. When I visited my patient next morning (the 17th), she was attempting to suckle her infant, quite free from pain, even on pressure; had several motions of more natural appearance during the night; pulse calm, about 108; made water freely; and felt a disposition to take some support. The turpentine was discontinued, as she felt almost well; but the abdomen being still swelled, it was ordered to be rubbed with a liniment composed of equal parts of Ol. Camph. and Sp. Terebinth. 18th. Passed a good night; had several hours sleep; stools and lochia natural; belly soft, and free from pain; skin moist; appetite returning; pulse 96. Was ordered some medicine of the tonic kind, and in a few days perfectly recovered.

This appeared to me a mixed case;—along with marked symptoms of peritonitis, the tensive pain over the eyes, and fermenting process in the stools, are considered symptoms of puerperal fever, some fatal cases of which had lately occurred hereabouts; but there was no vomiting at all in this instance. Whatever may be the nature of the turpentine's action on the system, it is certainly a most powerful and safe medicine, as was evinced in this case, by the entire removal of every bad symptom after its use. I trust more extensive experience of its effects will sanction it as a remedy in those distressing puerperal affections, particularly puerperal fever, hitherto so unmanageable, and almost uniformly fatal.

*April* 13, 1815. THOMAS ATKINSON.

